# 肺癌脑膜转移的研究进展

**DOI:** 10.3779/j.issn.1009-3419.2014.09.10

**Published:** 2014-09-20

**Authors:** 春华 马, 镕 姜, 金铎 李, 斌 王, 立伟 孙, 远 吕

**Affiliations:** 300060 天津，天津市环湖医院肿瘤介入科，天津市脑血管与神经变性重点实验室 Department of Intervention, Tianjin Huanhu Hospital, Tianjin Key Laboratory of Cerebral Vascular and Neurodegenerative Disease, Tianjin 300060, China

**Keywords:** 肺肿瘤, 脑膜转移, 诊断, 治疗, Lung neoplasms, Leptomeningeal metastases, Diagnosis, Treatments

## Abstract

脑膜转移是肺癌最严重的并发症之一，患者预后极差。脑膜转移患者临床表现缺乏特异性，主要表现为脑、脑神经、脊神经受累症状。目前诊断主要依据肿瘤病史、临床症状、增强核磁共振（magnetic resnance image, MRI）扫描和脑脊液细胞学检查。近年来新的检测方式在临床上不断涌现，明显提高了脑膜转移的早期发现率，但肺癌脑膜转移的综合治疗疗效仍不令人满意。本文就肺癌脑膜转移的病理生理、临床表现、诊断方法及治疗疗效的研究进展做一综述。

## Abstract (en)

Leptomeningeal metastases is one of the most serious complications of lung cancer, the patients with poor prognosis. Leptomeningeal metastasis in patients with lack specifcity of clinical manifestations. The main clinical performance are the damage of cerebral symptoms, cranial nerve and spinal nerve. The diagnosis primarily based on the history of tumor, clinical symptoms, enhance magnetic resnance image (MRI) scan and cerebrospinal fluid cytology. In recent years, new ways of detecting clinically, signifcantly increase the rate of early detection of leptomeningeal metastases. The effect of comprehensive treatments are still sad. The paper make a review of research progress in pathologic physiology, clinical manifestations, diagnosis methods and treatments of lung cancer with leptomeningeal metastases.

肺癌是我国目前最常见的恶性肿瘤之一，其发病率和病死率呈逐年上升趋势。肺癌患者预后不良的主要原因是局部复发或远处转移，其中脑膜转移(leptomeningeal metastasis, LM)的发现率约为5%^[1]^。肺癌脑膜转移患者预后极差，未接受治疗的患者中位生存期仅为4周-6周^[2]^。LM又称脑膜癌病，是指恶性肿瘤细胞通过血行转移、淋巴系统转移、脑脊液种植播散至脑膜或邻近肿瘤的直接侵犯脑膜的一种严重病变。临床表现为脑、脑神经和脊髓受损的症状。由于LM的临床表现复杂多样，缺乏特异性，一般的常规检查很难早期诊断，临床上很容易误诊和漏诊^[3, 4]^。近年来，随着肺癌治疗疗效的提高，肺癌患者生存期的延长，以及新的医学检查手段不断涌现，LM的发现率逐年增加。因此临床上应对LM给予足够的重视。本文就LM的病理生理、临床表现、诊断方法及治疗疗效的研究进展做一综述。

## 1 脑膜转移癌的病理生理

LM是肺癌的最严重并发症之一，约有5%的肺癌患者出现脑膜转移。LM的途径主要有^[3]^：血源转移到脉络膜丛血管而达蛛网膜下腔；血源转移到软脑膜而达蛛网膜下腔；转移到Batson静脉丛而达脑脊膜下腔；沿神经周围淋巴管及鞘逆行散布；血管周围淋巴管向心性蔓延；转移至颅骨再侵犯脑膜；各种原因包括医源性治疗检查手段，如化疗、手术、放疗或药物等可致血脑屏障破坏，也是肺癌脑膜转移的主要原因之一。进入蛛网膜下腔的瘤细胞通过脑脊液循环扩散，造成软脑膜弥漫性或多灶性浸润。浸润灶多发生在颅底部、脊髓背侧面和马尾。肿瘤细胞也可在脉络膜、室管膜等部位形成结节进而影响脑脊液循环和吸收，造成脑积水。亦可侵犯包绕神经的软脑膜，进而损伤神经。尸检可见脑膜转移患者脑组织水肿，脑回增宽，脑沟变窄，脑膜血管充血，脑脊髓膜浑浊增厚呈灰白色，切面可见脑室系统扩大。镜检可见癌细胞在脑脊髓软膜及蛛网膜下腔弥漫性播散或呈多灶性浸润，脑神经及脊神经根有不同程度的浸润，脑膜比脊髓膜重，癌细胞可沿血管周围间隙侵入脑皮质浅层，但在脑实质内不形成结节。

## 2 临床表现

LM的临床表现常因肿瘤细胞侵犯的部位不同而复杂多样，缺乏特异性，与脑实质、脊髓转移引起的症状以及治疗原发肿瘤出现的毒副反应很难鉴别。脑膜转移的主要临床表现有三种：脑实质受累及脑膜刺激症状，表现为头痛、呕吐、颈项强直、脑膜刺激征、精神状态改变、意识朦胧、认知障碍、症状性癫痫发作和肢体活动障碍等；脑神经受累症状，常见的受累脑神经有视神经、动眼神经、滑车神经、外展神经、面神经、听神经等，表现为视力下降、复视、面部麻木、味觉和听觉异常、吞咽和发音困难等；脊髓和脊神经根刺激症状，表现为节段性感觉缺损、肢体麻木、感觉性共济失调、腱反射减弱或消失、括约肌功能障碍、神经根性疼痛等^[3, 5]^。

## 3 诊断方法

目前，国内诊断LM多采用以下标准^[6]^：①有明确的肿瘤病史；②临床上有新发的神经系统症状和体征；③脑脊液细胞学检查阳性；④典型的核磁共振(magnetic resnance image, MRI)影像学表现，凡具备①、②项加上③或④项即可诊断。但脑脊液的细胞学检查发现肿瘤细胞仍是诊断LM的金标准^[2, 7]^。

### 3.1 脑脊液检查

脑脊液检查是诊断LM的关键，其中包括脑脊液常规检查、脑脊液细胞学检查、脑脊液免疫细胞化学检查、脑脊液肿瘤标志物检查和脑脊液循环肿瘤细胞检测(circulating tumor cells, CTCs)等。

#### 3.1.1 脑脊液常规检查

几乎所有的脑膜转移患者都存在脑脊液常规检查异常，但缺乏特异性。主要表现为不同程度的颅内压增高、白细胞数增多、蛋白质增多、氯化物正常或降低、葡萄糖降低，主要机制是肿瘤细胞浸润脑膜及其肿瘤代谢产物的化学刺激，血脑屏障的破坏和血管通透性增加，导致白细胞及蛋白渗出增加。葡萄糖降解产酸，在酸性环境下氯化物水平降低，持续性颅压升高，导致呕吐，亦可机体氯化物水平下降^[4]^。脑脊液中大量肿瘤细胞加速糖酵解和阻滞血糖通过血脑屏障，引起血糖含量降低。值得注意的是，一些亚急性或慢性脑膜炎也可引起上述脑脊液常规改变。

#### 3.1.2 脑脊液细胞学检查

脑脊液细胞学检查在脑膜转移的诊断中起着非常重要的作用，但首次腰椎管穿刺术检查脑脊液肿瘤细胞的阴性率在45%以上，第二次检查的阳性率可提高至80%，3次以上的检查无益于提高检查的阳性率^[2]^。脑脊液的细胞学阳性是诊断脑膜转移的金标准，但存在着一些问题^[2, 7-9]^：肿瘤细胞在腰穿留取的脑脊液样本中的出现存在相当大的随机性；在疾病早期由于脑脊液中肿瘤细胞数量少或者肿瘤细胞异型性小以及一些反应性间皮细胞的影响，很难早期有阳性发现；脑脊液细胞学检查主要从细胞形态上对异型性明显的肿瘤细胞做出快速诊断，但在细胞的性质及数量上无法做出判断；脑脊液取材量的限制、收集的脑脊液与肿瘤部位存在距离、检测的延迟均可导致脑脊液检查的敏感度降低。

#### 3.1.3 脑脊液免疫细胞化学检查

脑脊液免疫细胞化学检查能够弥补脑脊液细胞学检查在细胞性质鉴定方面的困难，提高L M的早期诊断率，亦可为寻找原发灶提供了有力依据。临床上常用的指标有癌胚抗原(carcinoembr yonic antigen, CEA)和上皮细胞膜抗原(epithelial membrane antigen, EMA)两项指标，CEA与EMA可作为上皮恶性肿瘤的重要标志物，二者联合应用在很大程度提高了对脑膜转移患者诊断的敏感度。窦春阳等^[10]^采用免疫荧光细胞化学技术和激光扫描共聚焦显微技术对20例脑膜癌病的脑脊液中EMA进行检测分析，结果提示脑脊液EMA阳性率可达90%，患者脑脊液免疫细胞化学检查能有效区分肿瘤细胞、软脑膜间皮细胞及反应性炎性细胞，但它亦有一定的局限性，如不同来源的肿瘤细胞标记物的交叉反应性和相对特异性，未分化或低分化肿瘤可能不表达抗原，以及脑脊液采集量的限制，不能同时做多种标记物检查等。

#### 3.1.4 脑脊液肿瘤标志物检查

肿瘤标志物(tumor marker, TM)是指在肿瘤发生和增殖过程中，由肿瘤细胞生物合成、释放或者是宿主对癌类反应性的一类物质，是肿瘤患者重要的检查指标。临床上常用于检测肺癌的肿瘤标志物有癌胚抗原(carcinoembryonic antigen, CEA)、神经原特异性烯醇化酶(neuron specific enolase, NSE)和细胞角蛋白19片断(cytokaratin 19 fragment, Cyfra21-1)。肺癌患者血清CEA水平明显高于正常对照组和良性肺病组患者，腺癌患者的阳性率明显高于鳞癌，非小细胞肺癌(non-small cell lung cancer, NSCLC)患者的阳性率明显高于小细胞肺癌。NSE是一种多肽酶类，其对小细胞肺癌的诊断有较高的敏感性和特异性，而对鳞癌和腺癌敏感性较低。Cyfra21-1是利用单克隆抗体识别的细胞角质蛋白19片段，在肺良性疾病和健康人群中含量很低，在鳞癌患者血清中的水平明显高于其它类型的肺癌患者。王鹏等^[11]^通过对35例肺癌脑膜转移患者进行血清及脑脊液肿瘤标记物检测分析，根据受试者工作曲线确定满足正确诊断指数最大值的临界点为脑脊液检测阳性判定标准，即CEA > 4.7 μg/L、NSE > 14.6 μg/L、CYFRA21-1 > 5.5 μg/L。CEA、NSE和CYFRA21-1的敏感性和特异性分别为91.4%、91.4%、51.4%和94.3%、82.9%、97.1%。三项指标任意1项阳性的诊断敏感性100%，三项指标同时阳性的诊断特异性100%。总结出脑脊液肿瘤标志物可以用于诊断脑膜转移，尤其对于那些细胞学和MRI检查难以确诊的患者，具有重要的临床意义。但脑脊液肿瘤标志物检测仍存在一些问题，如对原发肿瘤不能定性，以及脑转移、颅脑损伤、脑炎等对血脑屏障的破坏而影响检查结果等。

#### 3.1.5 脑脊液循环肿瘤细胞(circulating tumor cel l s, CTCs)检测

CTCs即自发或因诊疗操作由实体瘤或转移灶释放进入外周血循环的肿瘤细胞^[12]^。外周血CTCs检测在肺癌协助诊断、早期发现肿瘤的微转移、指导个体化治疗、评价治疗效果及预后方面具有重要的临床意义^[13, 14]^。CTCs属于非血源性上皮细胞，可通过富集技术及分析技术进行检测。目前国际上自动化最高的检测技术是CellSearch系统，该系统集免疫磁珠富集技术和免疫荧光技术于一体，具有较高的特异性、敏感性及可重复性^[15]^。国外研究^[2, 16]^表明，应用CellSearch系统检测乳腺癌、恶性黑色素瘤脑膜转移患者脑脊液中的循环肿瘤细胞，结果提示循环肿瘤细胞检测可能成为脑膜转移癌的诊断、患者随访以及化疗后疗效评价的方法。姜镕等^[17]^应用TM-iFISH技术检测5例肺腺癌脑膜转移患者7.50 mL脑脊液，共发现18个-1, 823个循环肿瘤细胞，提示该技术有可能成为新型诊断脑膜转移癌的方法，但该类技术在脑膜转移诊断中的应用尚处于初级阶段，其敏感性、特异性尚需要进一步的临床试验加以验证。

### 3.2 影像学检查

在LM的诊断中最有价值的影像学检查是脑部MRI增强扫描，其典型表现可以分为4种类型^[18]^：①脑积水：伴或不伴有脑膜或室管膜的强化；②硬脑膜-蛛网膜强化型：表现为颅骨内板大脑凸面连续的、粗的弧线形强化，不延伸至脑沟内；③软脑膜-蛛网膜型：表现为脑表面连续的、可延伸至脑沟内的细线状或结节状强化；④室管膜下强化。MRI增强扫描在脑膜转移诊断中的特异性几乎能够达到100%，但其存在65%的假阴性率和10%的假阳性率^[19]^。如感染性疾病、反应性脑膜增厚和非感染性脑膜炎等疾病亦可引起脑膜异常强化。当MRI增强扫描鉴别困难时还要结合临床症状、体征以及脑脊液检查，对于无法进行MRI增强扫描检查的患者，脑部CT增强扫描也可显示脑膜或转移病灶处强化作为参考，但常规平扫CT对脑膜转移的诊断价值不大^[20]^。

## 4 治疗方法

肺癌脑膜转移患者的预后极差，未经治疗的脑膜转移患者中位生存期仅为4周-6周，患者常死于进行性的神经功能破坏。目前LM治疗手段主要包括保守治疗、手术治疗、放射治疗、鞘内化疗、全身化疗和分子靶向治疗等。但上述治疗疗效并不理想，治疗相关毒副作用大，且无统一的治疗原则，经综合治疗后患者的中位生存期约4个月-6个月。治疗的主要目标是改善或稳定神经功能、延长生存时间、提高患者生活质量。

保守治疗主要是应用糖皮质激素及脱水药物，暂时缓解颅内高压症状，但需要同时对LM进行治疗，否则治疗的效果随时间延长而减弱，且对患者的生存期影响不大。

手术治疗常作辅助性治疗措施，如肺癌脑膜转移患者出现颅内压增高症状或脑室有扩大时，可行侧脑室-腹腔分流手术，以降低颅内压，缓解患者临床症状。另外，可放置经脑室Ommaya储液囊进而避免鞘内化疗所需的反复腰穿，通过Ommaya储液囊增加给药治疗的频率，维持脑脊液中化疗药物的治疗浓度以提高治疗疗效。Lin等^[21]^研究报道将Ommaya储液囊与侧脑室-腹腔分流管结合治疗了24例脑膜转移患者，中位无进展时间和中位生存期分别达到了14周和31周。Jung等^[22]^回顾性分析71例脑膜转移癌患者，结果发现局部放射治疗、鞘内化疗以及全身化疗能够改善患者生存期，而经外科治疗的脑积水患者(中位生存期5.7个月)与未经外科治疗的脑积水患者(中位生存期1.7个月)以及不合并脑积水患者(中位生存期2.3个月)的生存期无明显差异，研究结果表明外科治疗脑膜转移癌合并脑积水患者疗效欠佳。

放射治疗曾是LM常用的治疗方法之一，但放射治疗不能有效治疗整个蛛网膜下腔的转移瘤，进而影响临床疗效。全脊髓放射治疗易伴发严重的骨髓抑制，且易引发高病死率，目前临床上已经较少采取。Morris等^[23]^回顾性分析125例肺癌脑膜转移患者，所有患者的中位生存期为3.0个月，经全脑放射治疗的患者(*n*=46)与未经全脑放射治疗患者(*n*=59)生存期比较无统计学差异(*P*=0.84)。7例接受鞘内化疗患者的中位生存期为18个月(5个月-33个月)，9例表皮生长因子受体(epidermal growth factor receptor, *EGFR*)突变患者接受了EGFR-酪氨酸激酶抑制剂(EGFR-tyrosine kinase inhibitors, EGFR-TKI)药物治疗，中位生存期为14个月(1个月-28个月)，结果提示全脑放射治疗不能够提高患者的生存期。

鞘内化疗是LM的主要治疗方法之一，经腰穿将化疗药物直接注入蛛网膜下腔，能有效治疗肿瘤细胞沉积在脑膜所形成的瘤灶和脑脊液中的肿瘤细胞。Beauchesne^[24]^研究发现鞘内化疗可有效提高患者生存期，但鞘内化疗应用的药物是研究的重点。目前鞘内化疗可选择的药物很少，主要是甲氨蝶呤、阿糖胞苷和噻替哌3种。由于鞘内大剂量多次注射化疗药物，会产生较重的神经系统毒性，反复腰穿亦会增加患者的心理负担，且存在化学性脑膜炎及颅内感染风险。

随着新的化疗药物的不断涌现，能透过血脑屏障化疗药物有亚硝脲类药物如尼莫司汀和洛莫司汀，其他如甲氨蝶呤、顺铂、替尼泊苷、替莫唑胺、紫杉醇、培美曲塞等。Oechsle等^[25]^回顾性分析135例脑膜转移患者的治疗疗效，28%的患者接受全身化疗联合局部化疗，患者的生存期明显高于单纯局部化疗和放射治疗患者(5.6个月 *vs* 1.7个月)，结果表明全身化疗可延长患者生存期，其作用比局部化疗更加明显。Segura等^[26]^实验性应用替莫唑胺一线治疗脑膜转移癌19例，患者中位TP为28 d，中位OS仅为43 d，结果提示替莫唑胺在脑膜转移瘤治疗中的地位和作用有待于进一步探讨。

研究^[27]^证实EGFR在45%-70%的NSCLC中呈过度表达。EGFR-TKI在NSCLC治疗中取得较好的疗效，肺癌患者对EGFR-TKI的敏感性与EGFR基因突变密切相关^[28]^。Togashi等^[29]^研究15例EGFR突变的NSCLC脑膜转移患者应用厄洛替尼或吉非替尼治疗后脑脊液中的药物浓度及药物渗透率，结果发现吉非替尼的脑脊液含量为(3.7 ±1.9) ng/mL，药物渗透率为1.13%±0.36%，厄洛替尼脑脊液含量为(28.7±16.8) ng/mL，药物渗透率为2.77%±0.45%，研究结果表明EGFR-TKI的分子量较小并可少量通过血脑屏障。近年来逐渐应用分子靶向药物治疗NSCLC脑膜转移患者。国外研究^[30, 31]^应用大剂量厄洛替尼冲击治疗NSCLC脑膜转移，患者的临床症状、脑脊液变化及影像学表现均明显好转。关于EGFR-TKI治疗后复发的脑膜转移患者的治疗策略，Yuan等^[32]^应用培美曲塞联合大剂量吉非替尼(500 mg/d)治疗吉非替尼一线治疗后复发的脑膜转移患者1例，患者二线治疗至疾病进展时间达6个月。Hata等^[33]^应用厄洛替尼联合全脑放疗治疗1例吉非替尼治疗失败的脑膜转移患者，取得了较好的临床效果。此外，有研究^[34-36]^表明吉非替尼一线治疗失败的NSCLC脑膜转移患者应用厄洛替尼治疗仍可取得较好的疗效。由于上述研究样本量较小，EGFR-TKI类分子靶向药物治疗脑膜转移癌的临床疗效、给药方法以及一种靶向药物耐药使用另一种靶向药物的循证医学证据等问题尚需要临床多中心数据加以论证。

## 5 结论

综上所述，LM是肺癌的严重并发症之一，患者预后极差。患者临床表现缺乏特异性，主要表现为脑、脑神经、脊髓及脊神经受侵犯症状。脑脊液细胞学检查及增强MRI扫描作为脑膜癌诊断的主要手段，但二者早期诊断脑膜转移敏感性较差。脑脊液CTCs检测作为新的检测技术表现出良好的前景。脑膜转移的治疗方式主要有保守治疗、手术治疗、鞘内化疗、全身化疗和分子靶向治疗，治疗的目的主要是改善或者稳定患者的中枢神经功能，提高生活质量和延长生存期。随着新的化疗药物的不断涌现，化疗的地位和作用需要进一步的探讨。分子靶向治疗作为新兴治疗方式明显延长了*EGFR*基因突变患者的生存期，但其应用受病理类型及*EGFR*基因突变情况的限制。早期诊断脑膜转移并早期治疗能有效减少因病情进展而导致的神经功能损伤^[37]^，因此寻找一种更灵敏的方法来检测脑膜的潜在转移是需要解决的首要问题。另外，对于肺癌患者能否早期预防脑膜转移的发生以及最佳的治疗模式也是我们需继续研究与探索的问题。
